# Controlled Growth Cu_2_S Nanoarrays with High-Performance Photothermal Properties

**DOI:** 10.3390/nano13071260

**Published:** 2023-04-03

**Authors:** Huanran Miao, Yanlong Wu, Cheng Zhou, Zhimao Yang, Chuncai Kong

**Affiliations:** 1 Ministry of Education Key Laboratory for Non-equilibrium Synthesis and Modulation of Condensed Matter, School of Physics, Xi’an Jiaotong University, Xi’an 710049, China; 2 China Academy of Space Technology, Beijing 100094, China; long51641@126.com (Y.W.);

**Keywords:** Cu_2_S nanoparticles, photothermal, FDTD

## Abstract

The controlled growth of Cu_2_S nanoarrays was constructed by a facile two-step impregnation synthesis route. The as-synthesized Cu_2_S/CuO@Cu samples were precisely characterized in terms of surface morphology, phase, composition, and oxidation states. At the laser irradiation of 808 nm, Cu_2_S/CuO@Cu heated up to 106 °C from room temperature in 120 s, resulting in an excellent photothermal conversion performance. The Cu_2_S/CuO@Cu exhibited excellent cycling performance—sustaining the photothermal performance during five heating-cooling cycles. The finite difference time domain (FDTD) simulation of optical absorption and electric field distributions assured the accuracy and reliability of the developed experimental conditions for acquiring the best photothermal performance of Cu_2_S/CuO@Cu.

## 1. Introduction

Semiconductor nanomaterials have gained worldwide attention, due to their diverse designs and excellent physical and chemical performance [1]. Among them, chalcogenides possessing unique optical and electronic properties have been emerging as prominent research areas in materials science, chemistry, and nanotechnology [2,3]. Specifically, typical chalcogenides such as MoS_2_, Cu_2_S, WS_2_, TaS_2_, MoSe_2_ and WSe_2_ have been demonstrated to be suitable candidates in electronics [4], sensors [5], energy storage and conversion [6], biomedicine [7], and solar cell applications [8].

Photothermal materials play a crucial role in photothermal therapy [9], solar heat collection [10], photothermal seawater evaporation [11], and photothermal catalysis [12]. So far, noble metal nanomaterials [13], carbon based materials [14], semiconductors, and organic materials [15] have been used for photothermal applications. In particular, semiconductor photothermal materials offer simple preparation, low price, high stability, and easy functionalization. For instance, copper sulfide (Cu_2-x_S (0 ≤ x ≤ 1)) as a p-type semiconductor exhibited unique electronic and optical properties; the bandgap of Cu_2-x_S varies from 1.1 eV to 2.0 eV (depending on the atomic composition) [16]. As a plasmonic material, it absorbs near-infrared (NIR) light and converts it into heat due to d-d transition of copper ion and non-radiative relaxation [17]. The broad absorption in the NIR region has made Cu_2-x_S, such as CuS, Cu_2_S, and Cu_7_S_4_, great candidates in the photothermal field [18,19,20]. For instance, researchers have found that under irradiation of 980 nm, modified-CuS nanoflowers were able to attain a temperature of 63.1 °C within 5 min [18]. In particular, Cu_2_S has the features of low cost, abundance, recyclability, low toxicity, good stability, and intrinsic plasmonic properties [21], leading to uses in the fields of electronics, optoelectronics, photoelectrochemicals, biosensors, and photothermal materials. It has been found that the morphology of Cu_2_S can be directly controlled to satisfy the demands of different functions. For example, Cu_2_S with various morphologies has been reported, such as nanoparticles [22], nanosheets [23], hollow cage structures [24] and thin films [25]. Among them, Cu_2_S nanoparticles, which can be modified in size and morphology, show high photothermal conversion ability in the NIR region [26]. Wang et al. [27] prepared membranes embedded with Cu_2_S nanoparticles, which exhibited excellent photothermal conversion performance. Gu et al. [17] proposed a design of Cu_2_S/Cu mesh that shows a quick temperature rise from 28 °C to 44 °C in 1 min under the irradiation of a xenon lamp (1 kWm^−2^).

Generally, Cu_2_S nanoparticles are prepared by the impregnation method and solvothermal method, which often include heating and a long reaction time. Herein, we report the facile synthesis of Cu_2_S nanoparticles/CuO nanowires@Cu (Cu_2_S/CuO@Cu) using a two-step impregnation method. The reaction process was carried out at room temperature and lasted less than one hour. The as-prepared Cu_2_S/CuO@Cu demonstrated excellent photothermal conversion ability. Under the laser irradiation of 808 nm, the surface temperature of Cu_2_S/CuO@Cu heated up to 106.0 °C from room temperature. Moreover, the photothermal conversion process of Cu_2_S/CuO@Cu was also studied through numerical simulation—using the finite difference time domain (FDTD) of optical absorption and electric field distribution.

## 2. Materials and Methods

### 2.1. Materials

Cu foil (0.3 mm, 3 N) was purchased from Sinopharm Chemical Reagent. Sodium hydroxide (NaOH), ammonium persulfate (APS), nitric acid (HNO_3_), and sodium sulfide nonahydrate (Na_2_S·9H_2_O) were purchased from Aladdin Reagents. All these chemicals were analytical reagents and used without further purification.

### 2.2. Preparation of Cu_2_S/CuO@Cu

Cu_2_S/CuO@Cu was fabricated by the impregnation method. Initially, Cu foil (1 × 1 cm^2^) was ultrasonically washed in a 2 M HNO_3_ aqueous solution for 1 min and cleaned in ethanol and deionized water before use. The cleaned Cu foil was immersed in 2 M aqueous solution of NaOH and 0.125 M APS for 15 min, and then cleaned by ethanol and deionized water sequentially. It was dried in air at 60 °C to obtain Cu(OH)_2_@Cu, which was named as CC-0. Next, Cu(OH)_2_@Cu was immersed in an aqueous solution of Na_2_S·9H_2_O for 30 min, washed with ethanol and deionized water several times, and dried in air at 60 °C. As per the used concentration of Na_2_S·9H_2_O, i.e., 1 M, 0.5 M, 0.25 M, 0.1 M, the sample was named as CCC-1, 2, 3, 4.

### 2.3. Sample Characterization

The surface of Cu_2_S/CuO@Cu was investigated using a transmission electron microscope (TEM—Talos F200X, Thermo Scientific, Waltham, MA, USA) and scanning electron microscope (SEM—JEOL JSM-7000F, Akishima, Japan). X-ray diffraction (XRD) measurement was performed to explore the crystalline structure of the samples using a diffractometer (D8 ADVANCE, Bruker, Mannheim, Germany) with Cu Kα radiation (1.54178 Å). X-ray photoelectron spectroscopy (XPS) measurements were carried out to investigate the chemical composition of Cu_2_S/CuO@Cu using a Thermo Fisher ESCALAB Xi+ instrument equipped with a monochromatic Al Kα X-ray source operating at 400 W. The adventitious C 1s peak (284.8 eV) was used as the binding energy reference. Raman spectra of the synthesized samples were recorded by HR800 spectrometer with a 532 nm laser source. Visible-infrared (vis-NIR) spectroscopic investigation of the sample was conducted using a UV-vis spectrophotometer (U-4100, Hitachi, Japan).

### 2.4. Photothermal Test

A 4.24 W Xenon lamp (LR-ISP808/4000mW-BH81497) was used as the light source, and the power density irradiated on the sample was calibrated as 0.025–3 W/cm^2^. The temperature of the sample was measured by Fluke Thermography (Ti10-14070064). The experimental setup for the photothermal test is shown in Figure S1.

### 2.5. FDTD Simulation

FDTD solution was utilized to set up the structure, and to simulate the optical absorption and distribution of the electric field. A total-field/scattered field (TFSF) source (400–1200 nm) was used as an incident field into the simulation region. Perfectly matched layer (PML) boundary was utilized to calculate the absorption cross-section. In our calculations, we have kept the mesh resolution as 10 nm.

## 3. Results and Discussion

### 3.1. Characterization of Cu_2_S/CuO@Cu

**Figure 1:**
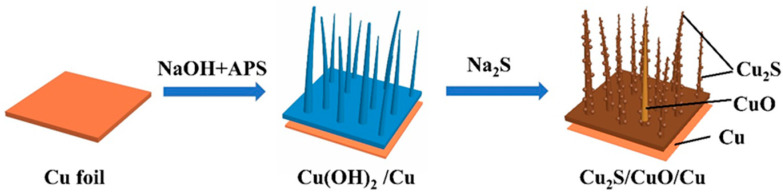
Schematic illustration of the fabrication process of Cu_2_S/CuO@Cu.

**Figure 2:**
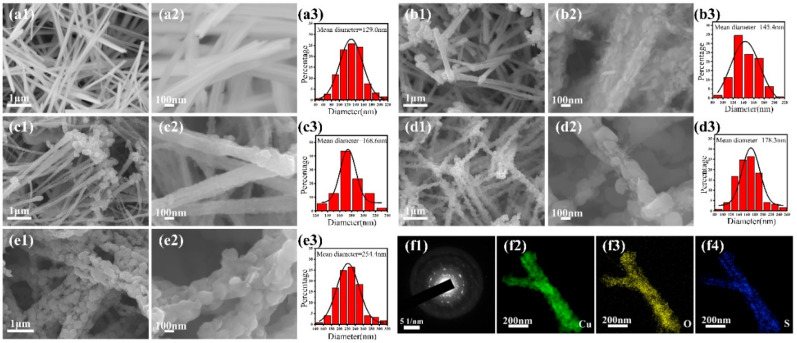
SEM images and size distributions of (**a1**–**a3**) CC-0, (**b1**–**b3**) CCC-1, (**c1**–**c3**) CCC-2, (**d1**–**d3**) CCC-3, (**e1**–**e3**) CCC-5; (**f1**) SAED and (**f2**–**f4**) elemental mapping of CCC-3.

The two-step impregnation procedure of Cu_2_S/CuO@Cu is schematically displayed in Figure 1. Under the effect of a strong alkaline solution, the crystallization of Cu(OH)_2_ nanowires occurs on the anodized Cu foil. The Cu(OH)_2_ nanowires were oxidized and formed CuO nanowire structures on the surface. The surface was further vulcanized to form Cu_2_S nanoparticles. The surface morphologies of Cu_2_S/CuO@Cu and Cu(OH)_2_@Cu are shown in Figure 2. From SEM images of Cu(OH)_2_ nanowires (Figure 2a1–a3), the wire shape of Cu(OH)_2_ is confirmed, and the nanowires exhibit size distributions of about 50–210 nm. Through vulcanization by Na_2_S, the Cu(OH)_2_ nanowires were converted to CuO nanowires covered with Cu_2_S nanoparticles. The components of Cu_2_S/CuO@Cu were further confirmed through elemental mapping (Figure 2f2–f4). With the growing concentration of Na_2_S, the diameter of the Cu_2_S nanowires is increased accordingly, as well as the size and number of Cu_2_S nanoparticles. The average Cu_2_S nanospheres diameters of CCC-1, 2, 3, 4 were measured to be 145.4 nm, 158.6 nm, 177.3 nm, and 254.5 nm, respectively.

**Figure 3:**
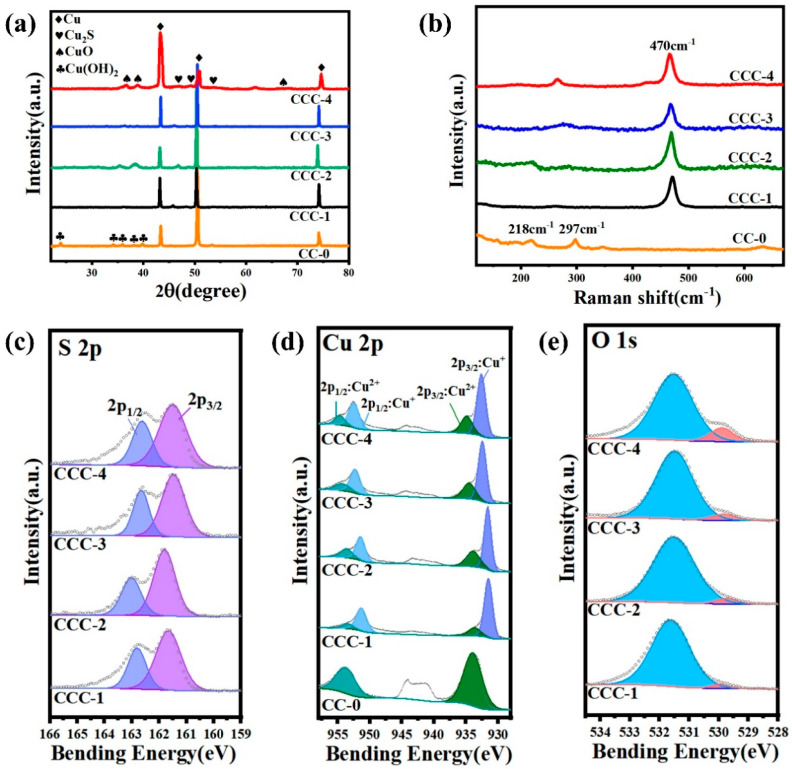
(**a**) XRD pattern, (**b**) Raman spectra, (**c**–**e**) XPS spectra of CCC-x and CC-0.

The crystalline phases of Cu_2_S/CuO@Cu and Cu(OH)_2_@Cu were identified by XRD. As shown in Figure 3, the diffraction peaks of Cu(OH)_2_@Cu confirm the existence of Cu(OH)_2_ nanowires and Cu substrates; the diffraction peaks of Cu_2_S/CuO@Cu are ascribed to the following phases: Cu_2_S—nanoparticles, Cu_2_O—nanowires and Cu—substrates, indicating that the Cu(OH)_2_ nanowires were oxidized to Cu_2_O nanowires and then vulcanized to Cu_2_S nanoparticles on the surface.

The chemical composition of Cu_2_S/CuO@Cu was analyzed using the outcomes of XPS performed on CC-0 and CCC-x (x:1–4). Survey of the XPS spectrum clearly demonstrates that Cu_2_S/CuO@Cu is mainly composed of Cu, S, and O elements. Additionally, Cu(OH)_2_@Cu was also studied by XPS for comparative analysis, confirming the presence of Cu and O elements. To analyze the chemical valence of Cu_2_S/CuO@Cu, high-resolution Cu 2p spectra were investigated. The Cu 2p spectrum of CC-0 exhibits two binding energies at 934.0 eV and 954.4 eV, which are associated with Cu 2p_3/2_ and Cu 2p_1/2_ of Cu^2+^. For the CCC-x sample, the Cu 2p spectrum exhibits four peaks, in which two peaks at 932.6 eV—Cu 2p_3/2_ and 952.6 eV—Cu 2p_1/2_ of Cu^+^, and two others peaking at 934.7 eV and 954.7 eV, are ascribed to Cu 2p_3/2_ and Cu 2p_1/2_ of Cu^2+^. The XPS spectrum of S 2p is deconvoluted into two peaks, resulting in S 2p_3/2_ at 161.5 eV and S 2p_1/2_ at 162.6 eV, respectively. In addition, the O 1s spectrum presents two deconvoluted peaks at around 529.8 eV and 531.6 eV, which correspond to the absorbed oxygen from the environment and O^2-^ binding with Cu^2+^ in CCC-x. Raman spectroscopy is an effective tool for studying molecular structure within nanostructures. Figure 3b shows the Raman spectrum of CCC-x; a strong and sharp band at 470 cm^−1^ is attributed to the vibrational mode of S-S bond stretching, confirming the Cu_2_S phase.

**Figure 4:**
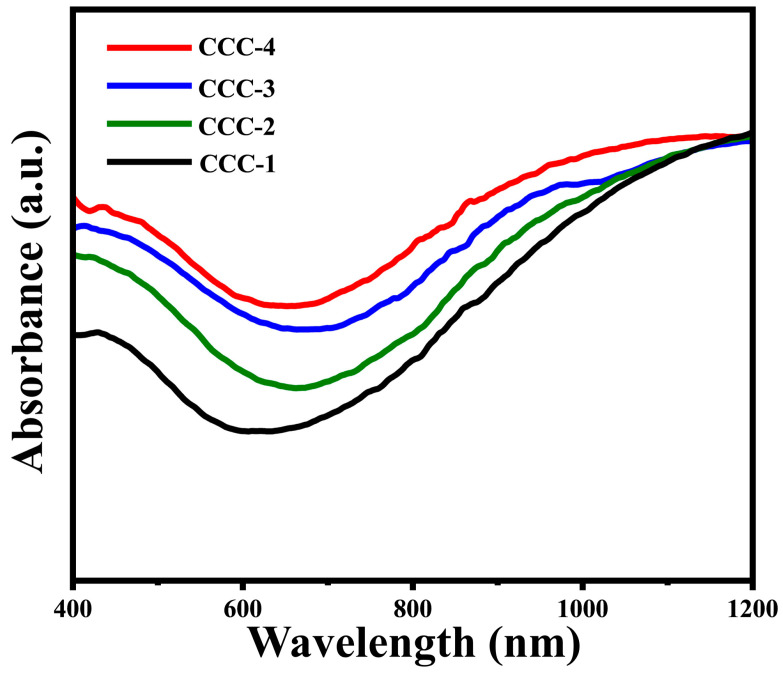
The optical absorbance of CCC-x.

In order to evaluate the optical characteristics of Cu_2_S/CuO@Cu, the vis-NIR absorption spectrum was collected, as shown in Figure 4. The absorption spectrum shows low absorption in the visible region and strong absorbance in the NIR region, which means that the Cu_2_S/CuO@Cu has a strong response to NIR irradiation. It could be clearly visualized that the absorption spectrum was red-shifted as the concentration of Na_2_S was reduced from 1.0 M to 0.1 M.

### 3.2. Photothermal Performance of Cu_2_S/CuO@Cu

**Figure 5:**
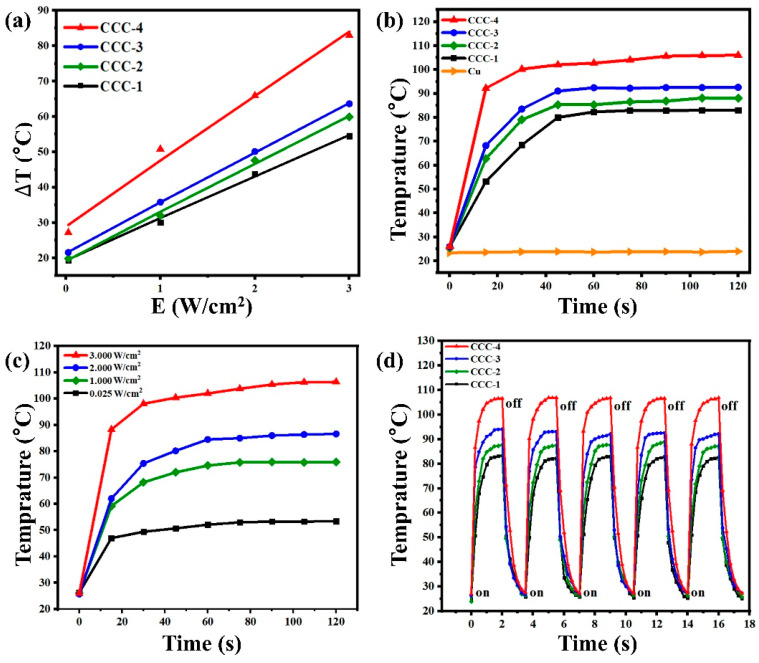
(**a**) Temperature gradient in CCC-x at 0.025, 1.0, 2.0, 3.0 W/cm^2^ laser irradiation, (**b**) temperature gradient in CCC-x and Cu at 3.0 W/cm^2^ laser irradiation for 120 s, (**c**) temperature gradient in CCC-4 at 0.025, 1.0, 2.0, 3.0 W/cm^2^ laser irradiation for 120 s, and (**d**) photothermal cycling test of CCC-x at 3.0 W/cm^2^ laser irradiation.

At 808 nm laser irradiation, the laser-induced photothermal effect on Cu_2_S/CuO@Cu was studied at room temperature. The experiment was conducted using four different power densities (0.025 Wcm^−2^, 1.0 Wcm^−2^, 2.0 Wcm^−2^, 3.0 Wcm^−2^) of laser irradiation, as shown in Figure 5. As the power density rises, the temperature increases gradually. At 0.025 Wcm^−2^, samples CCC-x heated up from room temperature to 44.6 °C, 44.9 °C, 46.7 °C, and 54.4 °C within 120 s, respectively. Further, at 3.0 Wcm^−2^, the samples heated up to 83.0 °C, 88.1 °C, 92.6 °C, and 106.0 °C within 120 s. In all cases, a steep temperature rise was observed within the first 20 s of irradiation, indicating the fast optical response capability of Cu_2_S/CuO@Cu. The temperature of sample CCC-4 reached 84.0 °C, which is much higher than previously reported [28]. Notably, pure Cu foil at the power density of 3.0 Wcm^−2^ shows only 0.6 °C temperature gradient in 120 s. The photothermal stability of Cu_2_S/CuO@Cu was demonstrated through five cycles of laser irradiation. As shown in Figure 5d, the photothermal capability remains the same after five cycles of heating and cooling (heating 120 s and cooling 90 s for one cycle); this result confirms the excellent photothermal stability of Cu_2_S/CuO@Cu.

### 3.3. Numerical Simulation of Cu_2_S/CuO@Cu

**Figure 6:**
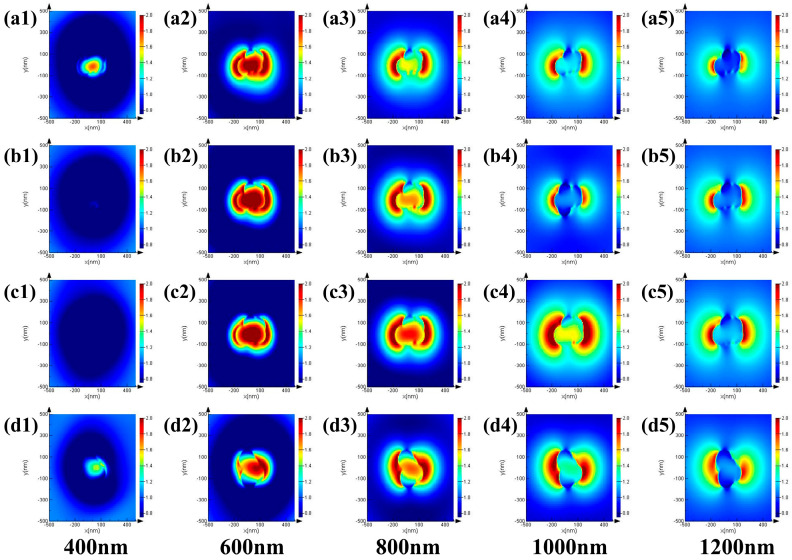
FDTD simulation of electric field distribution of (**a1**–**a5**) CCC-1, (**b1**–**b5**) CCC-2, (**c1**–**c5**) CCC-3, and (**d1**–**d5**) CCC-4 at different radiation wavelengths (400 nm, 600 nm, 800 nm, 1000 nm, and 1200 nm).

FDTD simulation of optical absorption and electric field distributions was studied to investigate the photothermal role of Cu_2_S/CuO@Cu. Figure S2 shows the comparison of the absorbance results obtained from FDTD simulation with the experimental measurements. The results are consistent, indicating the accuracy of the proposed model for purposes of investigating the electric field distribution of Cu_2_S/CuO@Cu. The structural model of Cu_2_S/CuO@Cu was constructed (Figure S3), and the electric field distributions (E/E_0_) of Cu_2_S/CuO@Cu were simulated at an incident light with a wavelength of 400–1200 nm. E and E_0_ were specified as reinforced electric field and incident electric field, respectively. Figure 6 shows the cross-sectional view of the electric field distributions of Cu_2_S/CuO@Cu.

As the simulated irradiation wavelength was increased from 400 nm to 1200 nm, the electric field distribution of Cu_2_S/CuO@Cu increased at first and then decreased. At 800 nm of irradiation, both the area and intensity of the electric field distribution were high; 808 nm laser light stimulated the strongest photothermal effect of Cu_2_S/CuO@Cu. Moreover, from the electric field distributions of CCC-x (x:1, 2, 3, 4), as the size of Cu_2_S nanosphere and nanowire size increased, the electric field distribution increased, leading to higher photothermal performance. Among all the Cu_2_S/CuO@Cu samples, CCC-4 demonstrated the highest electric field distribution numerically, which is consistent with the conducted experiment.

## 4. Conclusions

In summary, we have developed a simple two-step impregnation method to synthesize Cu_2_S nanoparticles grown on Cu-based substrate with controlled size by adjusting the concentration of Na_2_S. The as-synthesized Cu_2_S/CuO@Cu exhibited high performance in terms of photothermal conversion ability, with the maximum temperature raised to 106.0 °C from room temperature. The photothermal stability of Cu_2_S/CuO@Cu was proved through five heating-cooling cycles. Moreover, FDTD simulation of optical absorption and electric field distributions proved that the developed experimental condition enables the best photothermal performance of Cu_2_S/CuO@Cu.

## Data Availability

The data are not publicly available due to the relevant project regulations.

## Supplementary Materials

The following supporting information can be downloaded at: https://www.mdpi.com/article/10.3390/nano13071260/s1, Figure S1: Experimental setup for the photothermal experiment: (a) side view and (b) vertical view.; Figure S2: FDTD simulation (circle) and experimental data (solid line) of optical absorbance of CCC-x.; Figure S3: Structural model of the Cu_2_S/CuO@Cu in the FDTD simulation.
