# Supplementary material for: Controlled Growth Cu2S Nanoarrays with High-Performance Photothermal Properties
Source: Nanomaterials (Basel). 2023 Apr 3;13(7):1260. doi: 10.3390/nano13071260 (PMC10096858; doi:10.3390/nano13071260)
Supplement: Supplementary file 1 [file nanomaterials-13-01260-s001.zip › nanomaterials-2316774-supplementary.pdf]

Supplementary Materials

Controlled Growth Cu_2S Nanoarrays with High-Performance Photothermal Properties

Huanran Miao [1], Yanlong Wu [2], Cheng Zhou [2], Zhimao Yang [1] and Chuncai Kong [1,*]

[1] Ministry of Education Key Laboratory for Non-equilibrium Synthesis and Modulation of Condensed Matter, School of Physics, Xi'an Jiaotong University, Xi'an 710049, China

[2] China Academy of Space Technology, Beijing, 100094, China; long51641@126.com (Y.W.)

* Correspondence: kongcc@xjtu.edu.cn

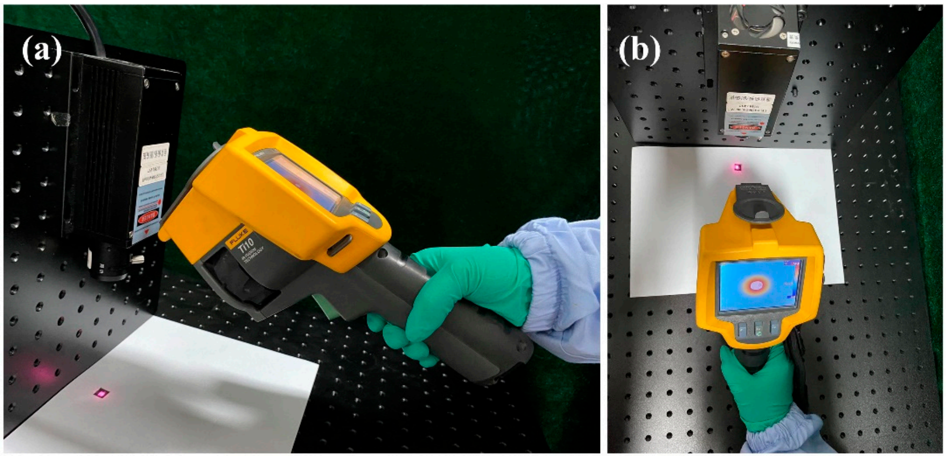

Figure S1. Experimental setup for the photothermal experiment: (a) side view and (b) vertical view.

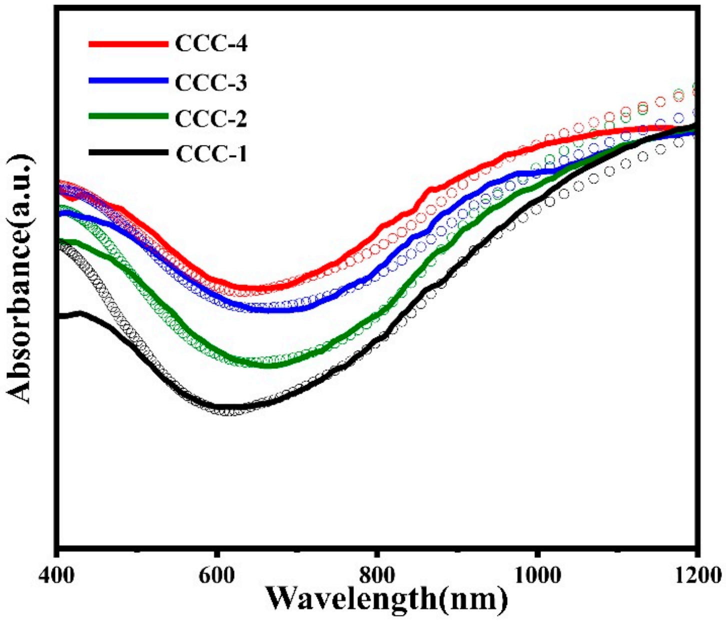

Figure S2. FDTD simulation (circle) and experimental data (solid line) of optical absorbance of CCC-x.

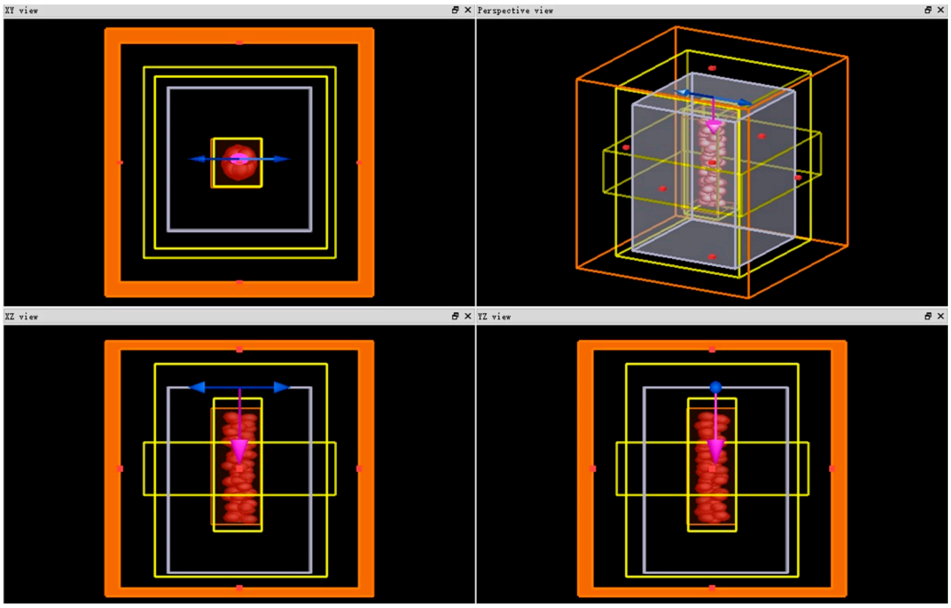

Figure S3. Structural model of the $Cu_2S/CuO@Cu$ in the FDTD simulation.
